# Elicitation of Roots and AC-DC with PEP-13 Peptide Shows Differential Defense Responses in Multi-Omics

**DOI:** 10.3390/cells11162605

**Published:** 2022-08-21

**Authors:** Marie Chambard, Mohamed Amine Ben Mlouka, Lun Jing, Carole Plasson, Pascal Cosette, Jérôme Leprince, Marie-Laure Follet-Gueye, Azeddine Driouich, Eric Nguema-Ona, Isabelle Boulogne

**Affiliations:** 1 University of Rouen Normandie, UNIROUEN, UFR des Sciences et Techniques, Glyco-MEV UR4358, Innovation Chimie Carnot, 76821 Mont-Saint-Aignan, France; 2 Fédération de Recherche Normandie-Végétal, FED 4277, 76821 Mont-Saint-Aignan, France; 3 University of Rouen Normandie, UNIROUEN, UFR des Sciences et Techniques, INSERM US 51, CNRS UAR 2026, HeRacLeS-PISSARO, IRIB, 76821 Mont-Saint-Aignan, France; 4 Agro Innovation International TIMAC AGRO, Plateformes Analytiques de Recherche, 35401 Saint-Malo, France; 5 University of Rouen Normandie, UNIROUEN, INSERM U1239 NorDIC, US51-UAR2026 HeRacLeS-PRIMACEN, 76000 Rouen, France; 6 Agro Innovation International TIMAC AGRO, Laboratoire de Nutrition Végétale, Pôle Stress Biotique, 35401 Saint-Malo, France

**Keywords:** root-associated cap-derived cells (root AC-DC), omics, PEP-13 elicitor, *Glycine max*

## Abstract

The root extracellular trap (RET) has emerged as a specialized compartment consisting of root AC-DC and mucilage. However, the RET’s contribution to plant defense is still poorly understood. While the roles of polysaccharides and glycoproteins secreted by root AC-DC have started to be elucidated, how the low-molecular-weight exudates of the RET contribute to root defense is poorly known. In order to better understand the RET and its defense response, the transcriptomes, proteomes and metabolomes of roots, root AC-DC and mucilage of soybean (*Glycine max* (L.) Merr, var. Castetis) upon elicitation with the peptide PEP-13 were investigated. This peptide is derived from the pathogenic oomycete *Phytophthora sojae*. In this study, the root and the RET responses to elicitation were dissected and sequenced using transcriptional, proteomic and metabolomic approaches. The major finding is increased synthesis and secretion of specialized metabolites upon induced defense activation following PEP-13 peptide elicitation. This study provides novel findings related to the pivotal role of the root extracellular trap in root defense.

## 1. Introduction

Plant roots are essential, complex, underground parts of plants. Roots are initially made by one primary root that, along with the developing plant, increases in size and shape to an array of individual roots that define a rooting system. This array of branched roots is organized into primary and lateral roots [1]. Roots can be subdivided into different functional zones, namely the root cap, meristematic, elongation, root-hair-abundant differentiation and finally, lateral root formation zones [2]. The root cap zone defines the foremost apical zone of the root and consists of different groups of cells, including columella cells and lateral root cap cells, the latter known for being programmed to separate from the root cap and be released into the external environment [3,4]. These detached cells are alternatively called border cells, border-like cells or root-associated cap-derived cells (root AC-DC) [3,4,5,6]. 

Roots interact with living organisms, including microorganisms [7]. These interactions are either beneficial or detrimental for plants. During negative or detrimental interactions, plant roots have developed means to protect themselves against these organisms. One of the plant cell natural barriers, or compartments, that pathogenic microorganisms have to circumvent during infection is the cell wall. The cell-wall compartment is mainly made of cellulose, hemicelluloses, pectins [8,9] and of so-called structural proteins such as hydroxyproline rich glycoproteins [10,11]. Together, these cell-wall components stand as a physical barrier against pathogen penetration in plant tissues. Changes in wall glycomolecule composition/organization can impact some pathogens’ ability to enter and progress within plant tissues. Extensins are cell-wall structural proteins that are known to crosslink in the wall, rendering the compartment more compact and less permeable [12,13]. 

Ingress of pathogens within this first layer of defense activates pattern-triggered immunity (PTI) when pathogen-derived molecules, or elicitors, are recognized by specific membrane receptors [14]. Several studies have investigated root defense responses during pathogen infection [15,16,17,18] or upon elicitation with pathogen-derived molecules [19,20,21,22,23,24]. The synthesis of defense-related proteins (e.g., pathogenesis-related (PR) proteins [25]) or specialized metabolites such as flavonoids [26] or phytoalexins [27] upon activation of plant immunity has been well described. Less described are cell wall compositional modifications and assembly/reorganization, which might take place upon infection and elicitation. Interestingly, upon elicitation of roots by plant elicitors, extensins have been shown to be synthesized and secreted in the walls [28]. Immunolocalization studies have also highlighted the importance of extensin glycosylation during this response to pathogenic attack [29,30,31]. Arabinogalactan proteins have also been shown to accumulate upon root elicitation [32,33].

Another key player of root defense is the RET system, made up of root AC-DC and root exudates (mucilage and other secretions), which together surround the root [6]. The RET, and especially root AC-DC, originate from the root tip. The root apical meristem (RAM), a cell division site from which root cells originate [34], produces newly divided cells that slowly move away from this zone and differentiate. Some of these cells will become root cap cells and finally (totally or partially) detach from the root to become part of the RET: the root AC-DC. 

The RET has two major functions: (I) attracting and trapping beneficial organisms, and (II) growth inhibition, repulsion or attraction, and trapping and immobilization of pathogens [5]. These functions are ensured in the RET thanks to its different components. 

Mucilage, as one of the materials secreted in the RET, has been shown to be able to trap pathogens such as *Nectria haematococca* Berk. & Broome in the RET of peas thanks to the presence of extracellular DNA in the mucilage [35]. In soybean RETs, the entry of zoospores of oomycete *Phytophthora parasitica* Dastur into root tissues was shown to be blocked by mucilage, inducing their lysis [36]. In *Pisum sativum* L. and *Brassica napus* L., AGP proteins in the mucilage attracted and stimulated the encysment of *Aphanomyces euteiches* Drechsler zoospores [32], and impacted *Pectobacterium atrosepticum* (van Hall) Gardan growth in potato root mucilage [33]. Specialized metabolites such as phenolic compounds are also released in mucilage, inhibiting pathogen growth, such as A. euteiches growth in pea RET mucilage [37]. 

Root AC-DC, besides producing and secreting mucilage components, respond upon elicitation and infection. Indeed, they have been shown to accumulate during defense responses [37,38] and are also able to produce reactive oxygen species, and reorganize their cell wall polymers as extensins or reinforce it with callose depositions [30,31].

The root system’s compartmentalized defense response has been well described [39], but the role of RET as a whole system in defense is not yet fully understood and deserves further research [40]. Each component of the RET, including the surrounding root, may have an organized, interconnected defense response. The objective of this work was to decipher the RET’s basal and induced defense responses using a single sampling, allowing us to investigate the RET system as a whole, namely the root, the root AC-DC and the root exudations. Soybean (*Glycine max* (L.) Merr)—a highly cultivated plant, mainly for human and animal nutrition, but also a plant known to release a high number of root AC-DC, associated with abundant mucilage [36]—was used as our model crop. A multi-omics study was developed, combining transcriptomic, proteomic and metabolomic data on this plant. The aim of this study was to unravel defense mechanisms, including physical, cell wall-related, but also induced mechanisms, on root, root AC-DC and their associated mucilage following the application of PEP-13, a microbial elicitor derived from the soybean pathogenic oomycete *Phytophthora sojae*.

## 2. Materials and Methods

### 2.1. Plant Material, PEP-13 Elicitation and RET Collection

*Glycine max* (L.) Merr. seeds from variety Castetis (La Dauphinoise, Vienne, France) were sterilized, cultivated and elicited as descripted in Chambard et al., 2021 [41]. Soybean RETs were collected aseptically by manual agitation of the roots in 400 µL of sterile water (for proteomic and metabolomic analysis) or in an RNA/DNA purification kit (Norgen, Thorold, Canada) lysis buffer for transcriptomic analysis. Root AC-DC were isolated from mucilage by centrifugation (10 min, 3000× *g*). Cell viability and cell separation from roots and mucilage were tested by microscopy and vital coloration with FDA (fluorescein diacetate, Sigma) to minimize contamination between root AC-DC, mucilage and root samples. Roots were crushed in liquid nitrogen. 

### 2.2. Transcriptomic Analysis

#### 2.2.1. Sample Preparation and Sequencing

RNA purification was performed per the RNA/DNA purification kit (Norgen) instructions; libraries were created with the NEBNext Single cell/Low input RNA Library Prep kit for Illumina (NEB); 75 bp single-read sequencing was done with a NextSeq500 from Illumina on three biological replicates with six roots for each replicate. 

#### 2.2.2. Data Analysis

FastQ files were uploaded on the Galaxy platform [42] and trimmed with Trimmomatic v0.36.6 [43], reads were aligned on the *G. max* genome (*Gmax* Wm82 a4 v1, Phytozome v13) with Hisat2 v2.1.0 [44], and gene count was done with HTSeq-Count v0.9.1 [45]. Differential expression of genes analysis was conducted with EdgeR v3.24.1 [46].

### 2.3. Proteomic Analysis

#### 2.3.1. Sample Preparation and Analysis

Four biological replicates of root AC-DC and roots collected from 250 plants were used for protein extraction after 2 freezing cycles and sonication in lysis buffer (7 M urea, 2 M thiourea, 4% CHAPS, 65 mM DTT, 25 mM Tris/HCl). The soluble proteins were recovered in the supernatant after centrifugation at 10,000× *g* for 30 min at 4 °C. 

Quantitative proteomic analyses were then performed with an LTQ-Orbitrap Elite (Thermo Scientific) coupled to an Easy nLC II system (Thermo Scientific), as previously described by Kentache et al., 2017 [47]. 

#### 2.3.2. Data Analysis

For protein quantification, data were analyzed using Progenesis LC–MS software (Nonlinear Dynamics, v4.0.4441.29989, Newcastle, UK) as previously described by Obry et al., 2014 [48]. Peptide identification was performed using Mascot (Matrix Science, version 2.2.04) against the database restricted to *Glycine max* from Uniprot. Mascot search results were imported into Progenesis. For each condition, the total cumulative abundance of the protein was calculated by summing the abundances of peptides. Proteins identified with fewer than 2 peptides were discarded. Only the proteins presenting a *p*-value ≤ 0.05 and varying by 1.5-fold in their average normalized abundances between different conditions were retained. 

### 2.4. Metabolomic Analysis

#### 2.4.1. Sample Preparation and Analysis

Four biological replicates of root AC-DC, mucilage and roots collected from 100 plants for each replicate were lyophilized and extracted with 70% MeOH (Optima LCMS grade, Fisher, Hampton, VA, USA), 29% H2O (Milli-Q, 18.2 MΩ·cm, Millipore, MA, USA) and 1% formic acid (LCMS grade, Fluka analytics, Munich, Germany). After extraction, samples were centrifuged, and the supernatant was collected for UPLC–MS/MS (ultra-performance liquid chromatography–tandem mass spectrometry). For UPLC–MS/MS, separation and detection were accomplished using an Acquity UPLC system (Waters, MA, USA) coupled to a Xevo G2-S QTof mass spectrometer (Waters) equipped with a LockSpray electrospray ionization (ESI) source. Sample separation was carried out by injecting 10 µL into a HSS T3 C18, 2.1 × 100 mm, 1.8 µm column (Waters) at a flowrate of 0.5 mL min-1, and the column oven was maintained at 40 °C. The mobile phases were composed of solvent A Milli-Q water containing 0.1% formic acid and solvent B acetonitrile containing 0.1% formic acid. Separation was achieved by the following gradient: 0–1 min at 98% A, 1–7 min from 98% to 0% A, maintained at 0% A to 9 min, 9–10 min from 0% to 98% A, and maintained at 98% until 12 min for column regeneration. MS analysis was carried out in positive and negative ionization modes with the following parameters: source voltage 0.5 kV (pos) and 2.5 kV (neg); cone voltage 40 V; source temperature 130 °C; desolvation gas temperature 550 °C; and desolvation gas flow 900 L/h. Mass spectra were acquired in MS^E^ mode from 50 to 1000 m/z at 0.1 s scan-1. Ramp collision energy was set at 25 to 40 V. Samples were injected in randomized order. A quality control (QC) sample was prepared from an equal mix of all collected samples. The QC sample was injected every 5 samples to assess system stability.

#### 2.4.2. Data Analysis

After acquisition, metabolomic data were processed using Progenesis QI software (v3.0, Waters). Metabolite abundance was calculated using peak area and was normalized to all compound ions. Identification was carried out using the PlantCyc database (v15.0.1) [49] with a mass tolerance of 10 ppm. For identified metabolites, experimental MS2 spectra were compared to the MS-DIAL reference MS/MS database (v14) [50] when possible, otherwise they were compared to the theoretical fragmentation spectrum. Multivariate analysis was performed on identified metabolites using EZInfo software (v3.0.3.0, Umetrics, Umeå, Sweden). Raw data were mean-centered and Pareto scaled [51,52]. Partial least squares discriminant analyses (OPLS-DA) [51,53] were carried out to classify samples with and without PEP-13 elicitation. The influence of each metabolite on the classification was calculated by the variable influence on projection (VIP) [51,53]. Metabolites with VIP > 1 have an above-average influence. In this study, only metabolites with VIP > 2 were kept for further analysis. For metabolites detected both in positive and negative modes, the one with the higher mean abundance was kept. Heatmaps were generated using Morpheus (https://software.broadinstitute.org/morpheus (accessed on 1 October 2020)). For a better understanding of metabolic regulation induced by PEP-13 elicitation, pathway analysis was performed using the open-source software MetaboAnalyst 5.0 [54]. For each pathway, the *p*-values of metabolite set enrichment analysis and pathway impact of topology analysis were calculated using KEGG database [55].

## 3. Results and Discussion

In this study, roots and root AC-DC transcriptomes and proteomes were first compared in the absence of any eliciting agent. This first experiment was designed to inform us of the possible specialization of one of these two compartments in the basal state condition. Subsequently, roots and root AC-DC were treated with the PEP-13 peptide elicitor in order to compare the transcriptome, proteome and metabolome obtained following elicitation on roots, root AC-DC and mucilage. 

### 3.1. A Picture of Basal State in Root Cells and Root AC-DC

**Figure 1:**
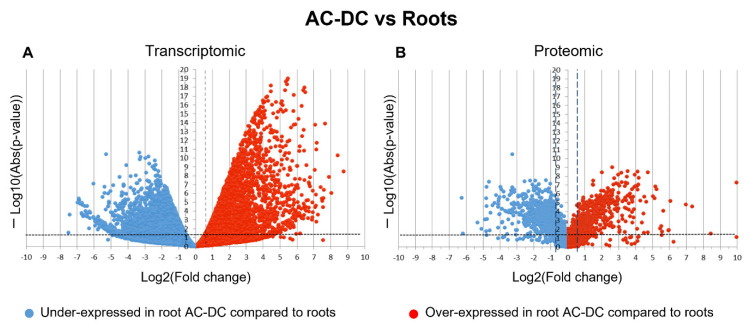
Differential expression analysis of genes (**A**) and proteins (**B**) in root AC-DC and roots. These volcano plots were obtained from transcriptomic (**A**) and proteomic (**B**) data. Blue points correspond to genes (**A**) and proteins (**B**) under-expressed in root AC-DC compared to roots. Red points correspond to genes (**A**) and proteins (**B**) over-expressed in root AC-DC compared to roots. The number of under- and over-expressed genes and proteins for each method appears in blue (under-expressed) and red (over-expressed) squares. DEG = differentially expressed genes; DEP = differentially expressed proteins (fold change ≤ 0.66 or fold change ≥ 1.5; *p*-value ≤ 0.05).

The first step for investigating the basal defense mechanisms of root AC-DC was to detect differentially expressed (FC ≥ 1.5; *p*-value ≤ 0.05) genes (DEG) and proteins (DEP) between roots and root AC-DC in non-elicited conditions (Figure 1). 

Differential analysis shows a high amount of differentially expressed genes and proteins. Indeed, 7774 DEG and 1215 DEP were found in our proteomic and transcriptomic studies. These results might indicate a difference between transcripts and proteins found in roots and in root AC-DC in constitutive conditions. For transcriptomic data, under-expressed genes (3779) seem to have a slightly lower quantity than over-expressed genes (3995) in root AC-DC. For proteomic data, under-expressed proteins (719) are found in a higher quantity than over-expressed proteins (496) in root AC-DC. Despite this different number of differentially expressed genes and proteins, transcriptomic and proteomic data showed similar main biological processes, including cellular and metabolic processes, response to stimulus, localization, biological regulation, reproductive and developmental processes, and biological processes involved in interspecies interaction between organisms. Some pathways, such as immune system processes (mainly kinase signaling and salicylic acid signaling pathway), rhythmic processes (mainly blue light response) and nitrogen utilization, were specifically found in roots. The cellular detoxification pathway (mainly corresponding to peroxidases) was specifically found in root AC-DC. These results suggest that soybean roots and root AC-DC have similar main biological processes, but express different transcripts and proteins. Root AC-DC can therefore be considered a specific compartment in the root system with its own regulation. 

**Figure 2:**
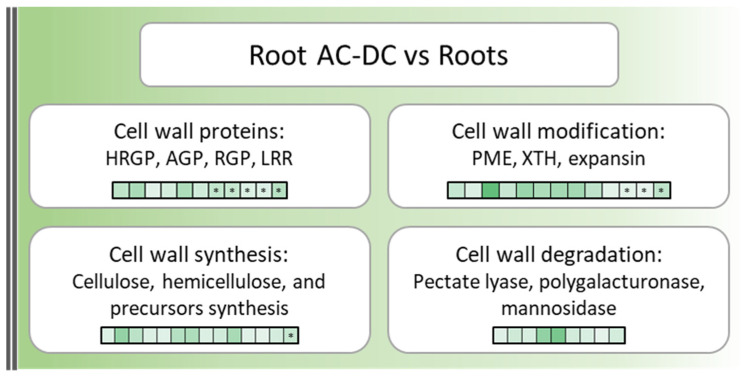
Cell wall over-expressed genes and proteins in root AC-DC compared to roots in constitutive conditions. Each square corresponds to a gene or protein (squares with *), dark green squares correspond to highly overexpressed genes or proteins compared to roots, light green squares correspond to lowly overexpressed genes or proteins in root AC-DC compared to roots (fold change ≤ 0.66 or fold change ≥ 1.5; *p*-value ≤ 0.05).

Cell walls and RET are essential for root defense [5]. They can act constitutively as a physical barrier, making up the first layer of defense. Thus, cell wall genes and proteins over-expressed in root AC-DC in comparison to roots in constitutive conditions (Figure 2 and Table S1) were investigated.

A total of 38 genes and 9 proteins over-expressed (FC ≥ 1.5; *p*-value ≤ 0.05) in root AC-DC compared to roots were found to be involved in cell wall biological processes. Among these genes and proteins, 20 cell wall enzymes involved in cell wall component synthesis (cellulose, hemicellulose, reversibly glycosylated polypeptides (RGP) and precursors) were found, including 9 involved in cell wall component degradation (pectate lyases and mannosidase). Cell wall proteins such as hydroxyproline-rich glycoproteins (HRGP) and expansins were found. HRGP (AGPs and extensins) are a family of proteins involved in plant defense thanks to diverse actions such as cell wall strengthening and agglutination of bacteria and zoospores [56]. Within the HRGP family, no differentially expressed extensin genes and proteins that are known for their roles in plant defense [29,57] were found. Thus, AGPs, which are known to be involved in many cell processes, such as embryogenesis, pollen tube growth and programmed cell death, but also in plant–microorganism communications and protection against biotic stresses [11,58] were detected. They also have been described in pea and rapeseed as chemoattractant proteins on *Aphanomyces euteiches* zoospores [32,59]. Regarding expansins, they are known to be involved in cell wall loosening, leading to different biological processes such as cell growth, root hair formation and elongation, cell wall remodeling during mycorrhiza symbiosis and even enhancing resistance to biotic and abiotic stresses upon transgenic expression in tobacco and Arabidopsis [60,61]. 

Enzymes involved in cell wall component modifications, such as xyloglucan endotransglucosylase/hydrolase (XTH) and pectin methylesterases (PME), were also found. XTH is responsible for xyloglucan polymer modifications, leading to the formation of a xyloglucan and cellulose network [62]. This network seems to be essential for mucilage strengthening and root AC-DC attachment and functioning [63]. PME catalyses pectin demethylesterification and is involved in different mechanisms, such as cellular adhesion, stem elongation and even cell wall strengthening, at a basic pH and in the presence of Ca^2+^ ions [64]. PME is also responsible for root AC-DC detachment from the root tip [65], and therefore it is not surprising to still find them in newly detached root AC-DC.

These findings indicate that roots and root AC-DC metabolism seem to both be constitutively oriented into nitrogen metabolism, MAPK (mitogen-activated protein kinase) signaling and blue light response. However, root AC-DC metabolism seems to be also constitutively oriented into cell wall synthesis and modification, as well as mucilage strengthening and defense or chemoattracting protein synthesis, resulting in RET function reinforcement. However, the cell wall is not only a constitutive defense barrier, it can also have induced defense responses upon infection and elicitation in the form of callose deposition or cell wall reorganization and strengthening [28,29,31]. 

### 3.2. Induced Defense Mechanisms upon PEP-13 Elicitation

In order to investigate the induced defense response of root AC-DC in the RET, the peptide elicitor PEP-13 was directly added to the culture medium, resulting in a 5-day elicitation. Then, the differential expression of genes and proteins was analyzed. 

#### 3.2.1. Transcriptomic and Proteomic Analysis of Induced Defense Response in the RET

**Figure 3:**
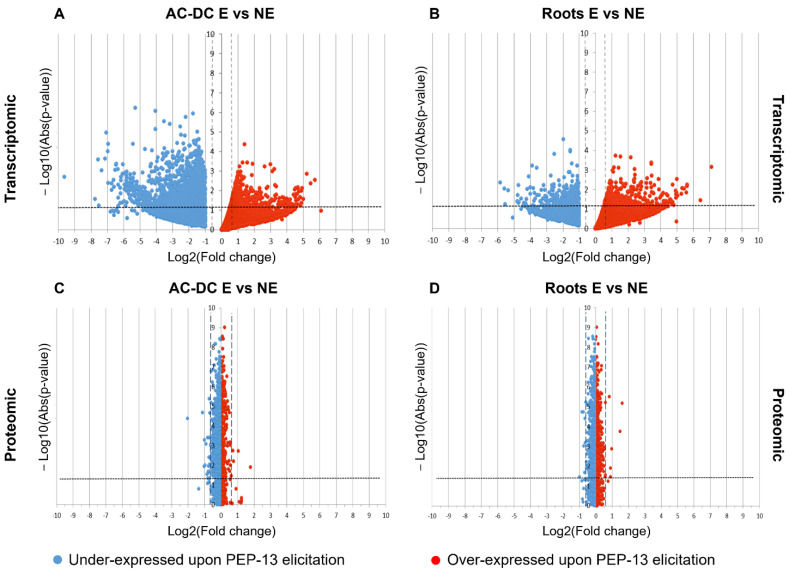
Differential expression analysis of genes (**A**,**B**) and proteins (**C**,**D**) upon PEP-13 elicitation in roots (**B**,**D**) and root AC-DC (**A**,**C**). These volcano plots were obtained from transcriptomic (**A**,**B**) and proteomic (**C**,**D**) data. Blue points correspond to genes and proteins that are under-expressed upon PEP-13 elicitation. Red points correspond to genes and proteins that are over-expressed upon PEP-13 elicitation. The number of under- and over-expressed genes and proteins for each method appears in blue (under-expressed) and red (over-expressed) squares. DEG = differentially expressed genes; DEP = differentially expressed proteins (fold change ≤ 0.66 or fold change ≥ 1.5; *p*-value ≤ 0.05).

Differential expression analysis show 2295 DEG in root AC-DC, and only 553 DEG in roots (Figure 3A,B). Proteomic results show 31 DEP in root AC-DC and 13 DEP in roots, which is low compared to transcriptomic results (Figure 3C,D). 

The higher number of DEG and DEP found in root AC-DC might indicate a larger response of these cells to PEP-13 elicitation than that of roots. A more accurate analysis of DEG and DEP (Table S2) allowed us to observe that in roots, over-expressed genes and proteins in response to PEP-13 elicitation are involved in signaling processes and are mainly G proteins (GLYMA_19G130700; GLYMA_07G085000; GLYMA_09G209900), which are involved in many processes such as biotic and abiotic stress response, symbiosis and nitrogen-use efficiency [66]. Over-expressed genes and proteins are also involved in redox mechanisms (GLYMA_04G079200; GLYMA_03G151500), ethylene synthesis (GLYMA_13G147600; GLYMA_09G243500; GLYMA_03G221600), cell wall metabolism (GLYMA_09G236200; GLYMA_05G190300), proteolysis (GLYMA_03G254500; GLYMA_02G046400), effector detection (GLYMA_16G033900; GLYMA_09G020700) and defense. Among defense genes and proteins, a kiwellin protein (glyma13g39890.1) was found, as well as two PR-proteins (GLYMA_16G033900; GLYMA_09G020700) and a chitinase (GLYMA_02G007400). Kiwellin proteins have been found to play a defense role against pathogens such as *Ustilago maydis* (DC.) Corda [67] and *Phytophthora infestans* (Mont.) de Barry [67]. Genes coding a pectinesterase (GLYMA_05G190300) and an expansin (GLYMA_09G236200), which are involved in root AC-DC detachment and cell wall loosening [61,65], were also found. Upon PEP-13 elicitation, soybean roots seem to activate defense responses (ethylene synthesis, kiwellin, PR-proteins and chitinase) and root AC-DC detachment. Indeed, it has been shown that root AC-DC quantity increases significantly in pea RET during infection with the oomycete *Aphanomyces euteiches* Drechler [37]. Thus, a similar root AC-DC increase might not be surprising in response to another elicitation by a *Phytophthora*-derived MAMP (microbe-associated molecular pattern). 

In root AC-DC, over-expressed genes and proteins in response to PEP-13 elicitation (Table S3) are involved in signal processes (GLYMA_07G085000; GLYMA_06G183500), ethylene synthesis (GLYMA_13G147600; GLYMA_13G218200), transport processes such as Golgi complex internal transport (Got1-like family protein; GLYMA_18G299400) and a PH domain-containing protein (GLYMA_11G244600) involved in sterol transport. Genes occurring in developmental processes—for example, genes coding NAC-domain proteins (GLYMA_16G151500; GLYMA_08G031900)—were detected. These genes are involved in different processes such as stress responses, hormone signaling, organ formation and development [68,69,70]. A gene coding for a patatin protein (GLYMA_06G037900), a plant storage protein with antimicrobial and phospholipase activities [71] was detected. The phospholipase activity of patatin and other over-expressed phospholipase genes (GLYMA_15G023500; GLYMA_15G152100) might release lipids from membrane degradation (diacylglycerol, phosphatidic acid, lysophospholipides, etc.) recognized as DAMPs (damage-associated molecular patterns) and lead to MAPK cascade activation, cytoplasmic pH changes, and PR gene regulation [72,73]. An osmotin protein (glyma05g38110.1) known as PR-5 was also over-expressed; it is involved in defense mechanisms against various pathogens, including oomycetes [74,75]. Some other over-expressed genes leading to DAMP recognition have been found, such as a germin-like protein coding gene (GLYMA_10G139800) and a trehalose phosphate synthase coding gene (GLYMA_12G234200). Germin-like proteins synthetize H_2_O_2_ from reactive oxygen species [76] produced by peroxidases (GLYMA_13G106400; GLYMA_14G201700), and trehalose plays a role in growth control and biotic and abiotic stresses resistance [77]. Cell wall over-expressed genes were also detected, such as pectin methylesterases (GLYMA_08G147900; GLYMA_19G231400), which is involved in cell adhesion, root AC-DC detachment, and cell wall reinforcement [64,65]; cellulose synthase (GLYMA_03G217500) and XTH coding gene (GLYMA_18G003200). XTH (xyloglucan endotransglucosylase/hydrolase) enzymes are known to act on xyloglucan polymers, enabling the formation of a xyloglucan-cellulose based network [64] that might be essential for maintaining mucilage cohesion and root AC-DC linking [65]. A protein involved in lignin synthesis (glyma18g38670.1) was also found. Lignins were found to participate in cell wall reinforcement upon oomycete *P. sojae* infection [78,79]. 

Thus, upon PEP-13 elicitation, root AC-DC seem to activate PTI (peroxidases, ethylene, kinases and PR-5 synthesis) and the production of DAMPs (trehalose, patatin and germin-like synthesis). This DAMP production might allow the reinforcement of defense mechanism activation of the whole RET. Another mechanism involved in response to PEP-13 elicitation might be mucilage reinforcement (cellulose and XTH synthesis leading to a xyloglucan–cellulose network). 

In root AC-DC, a high number of genes and proteins seem to be under-expressed in response to PEP-13 elicitation. Down-regulated genes were detected from different pathways, such as cellular processes, metabolic processes, biological regulation, cellular component organization and biogenesis. The most important negative fold change (Log(FC) = −9.6) corresponds to a cellulose synthase coding gene (GLYMA_06G069600). Down-regulated pectin esterase coding genes (GLYMA_05G236800; GLYMA_08G033000) were also detected, as well as a polygalacturonase coding gene (GLYMA_19G006200) and other cell-wall related genes (GLYMA_15G037700; GLYMA_11G141300; etc.). Thus, PEP-13 elicitation seems to have an inhibitory effect on genes involved in cellulose synthesis, pectin methyl esterification and degradation and widely on some cell wall synthesis and modification pathways. Among the most down-regulated genes, a DNA polymerase (GLYMA_18G009300), ribosomal proteins (GLYMA_02G079000; GLYMA_18G007500; GLYMA_10G006200; etc.) and a gene coding for a GRAM-domain-containing protein (GLYMA_08G281400) were found. These proteins are responsive to abscisic acid, and in rice, GRAM domain proteins are known for their role in plant–rhizobacteria interactions and abiotic stress responses [80]. 

In order to complete these transcriptomic and proteomic studies and to have access to the mucilage compartment, metabolomic analysis was conducted on our samples.

#### 3.2.2. Metabolomic Analysis of Induced Defense Response in the RET 

Untargeted metabolomic analysis enabled us to analyze metabolic changes that would have taken place in roots, root AC-DC and in the RET mucilage. 

**Figure 4:**
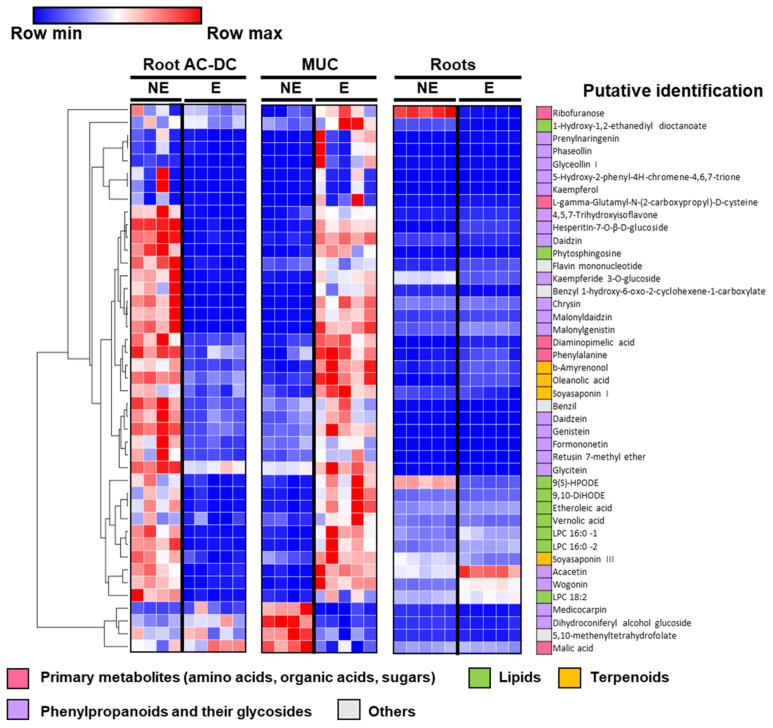
Heatmap of most-discriminative metabolites upon PEP-13 elicitation. Metabolite abundances were analyzed using UPLC–QTOF–MS–MS. Multivariate supervised classification OPLS-DA was established for each compartment (root AC-DC, MUC and roots) to discriminate between control (NE) and PEP-13 elicited (E) samples. The heatmap shows the peak abundance of all metabolites with a VIP > 2 in root AC-DC and Muc. The maximum abundance in each row is shown in red and the minimum in blue. MUC = mucilage; AC-DC = root-associated cap-derived cells; NE = non-elicited (control); PEP = PEP-13 elicited.

In order to more precisely investigate metabolomic differences in response to PEP-13 elicitation, supervised multivariate classification OPLS-DA was carried out for each compartment (roots, root AC-DC and mucilage) between samples with and without PEP-13 elicitation (Figure S1). The importance of each metabolite to discriminate PEP-13 elicitation effects was evaluated with VIP (variable importance on projection) in each respective classification. All metabolites with a VIP > 2 in root AC-DC and in mucilage were summarized in a heatmap to show the most discriminative metabolites upon PEP-13 elicitation (Figure 4). In total, this list contains 43 metabolites, out of which 25 also have a VIP > 2 in roots.

Among the 43 most discriminative metabolites (Figure 4), almost half (21 metabolites) correspond to phenylpropanoid compounds. In the rest of the metabolites, nine lipids, four terpenoids and five primary metabolites—including amino acids, organic acids and sugars—were found. 

In root AC-DC, most discriminative metabolites were mainly found in the non-treated conditions and seems to sharply decrease upon PEP-13 elicitation. Conversely, in mucilage, the same discriminative metabolites are present in lower quantities in control conditions, and increase significantly upon PEP-13 elicitation. Remarkably, the data suggest a transfer of metabolites from root AC-DC into the mucilage in response to PEP-13 elicitation. This change of compartment might involve mobilization and secretion of metabolites that are produced by root AC-DC. 

The root defense response seems to be very different from the RET (mucilage and AC-DC) defense response regarding these metabolites. Indeed, the majority of metabolites with a VIP > 2 in root AC-DC and mucilage do not seem to significantly increase or decrease upon elicitation. 

Among the phenylpropanoid compounds, daidzein and genistein were found; they are involved in NOD gene activation and nod factor secretion [81], and also attract and induce rapid encystment of *P. sojae* zoospores via chemotaxis [82]. In control conditions, these compounds are found mainly in root AC-DC, and upon elicitation, they are mainly found in mucilage samples. This change of compartment induced by PEP-13 elicitation might allow the RET to attract and immobilize *P. sojae* zoospores. 

Our data show that glyceollin I is found in mucilage upon elicitation by *P. sojae* cell wall peptide elicitor (PEP-13). Glyceollin I, a daidzein derivate [83], is a well-known phytoalexin involved in soybean resistance to *P. sojae* infection, whose production is also induced by *P. sojae* cell wall glucan elicitor [27]. 

Among terpenoid compounds, soyasaponins I and III were detected. Plant saponins are involved in plant defense against pathogens, herbivores and insects [84], and have been found to be secreted in soybean exudates [85]. The change of compartment of these compounds (daidzein, genistein and soyasaponins) might indicate that they are released by root AC-DC into the mucilage as part of the defense mechanisms of soybean RET.

In roots, these discriminative metabolites seem not highly differentially expressed between constitutive and elicited conditions, except a few of them such as ribofuranose, which is involved in the pentose phosphate pathway [86]; wogonin and acacetin, two flavonoid compounds (74); and 9(S)-HPODE, involved in linoleic acid metabolism [87] (Figure 4).

**Figure 5:**
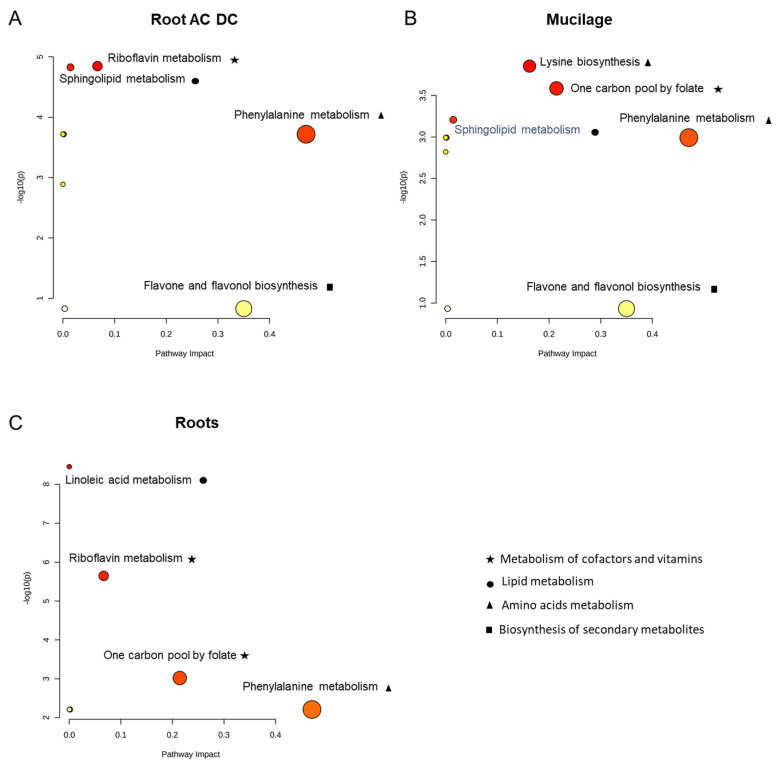
Pathway analysis of root, root AC-DC and mucilage upon induced defense response with PEP-13. In root AC-DC (**A**), mucilage (**B**) and roots (**C**), pathways are represented according to their impact value (pathway impact) and their *p*-value (−log10(*p*)). Pathways with impact value > 0.1 and −log10(*p*) > 1.3 are considered to be impacted.

A pathway analysis was also performed in root, root AC-DC and mucilage with all the VIP > 2 metabolites (Figure 5) using MetaboAnalyst, enabling us to determine a topology analysis-based impact value and enrichment analysis-based-*p*-values for each pathway.

In root AC-DC (Figure 5A), only one pathway was clearly impacted: phenylalanine metabolism. This observation corresponds to the high detection of phenylpropanoid compounds (Figure 4), which are derived from phenylalanine [88,89]. 

In mucilage (Figure 5B), impacted pathways are lysine biosynthesis, one carbon pool by folate, and phenylalanine metabolism. The one carbon pool by folate pathway impact seems to correspond to the metabolite 5,10-methenyltetrahydrofolate (Figure 4), which is found at high levels in control conditions in mucilage and decreases upon elicitation. This pathway is involved in genomic DNA methylation, and its disruption has been shown to promote enhanced disease resistance to *Pseudomonas syringae* DC3000 in *Arabidopsis thaliana* [90]. This pathway and the phenylalanine pathway were also found to be impacted in roots (Figure 5C).

For each compartment—roots, root AC-DC and mucilage (Figure 5)—the most-impacted pathway is the phenylalanine pathway: a primary metabolism pathway. This observation led us to analyze primary metabolites involved in PEP-13-induced defense mechanisms.

#### 3.2.3. Focus on Primary Metabolism

In order to better understand root AC-DC metabolism, primary metabolites involved in root AC-DC-induced defense response were analyzed. Indeed, these metabolites are key players indicating metabolism changes. Only five primary metabolites with a VIP > 2 were found (Figure 5): ribofuranose; L-gamma-glutamyl-N-(2-carboxypropyl)-D-cysteine, a cysteine derivative; diaminopimelic acid, a lysine precursor; phenylalanine and malic acid.

**Figure 6:**
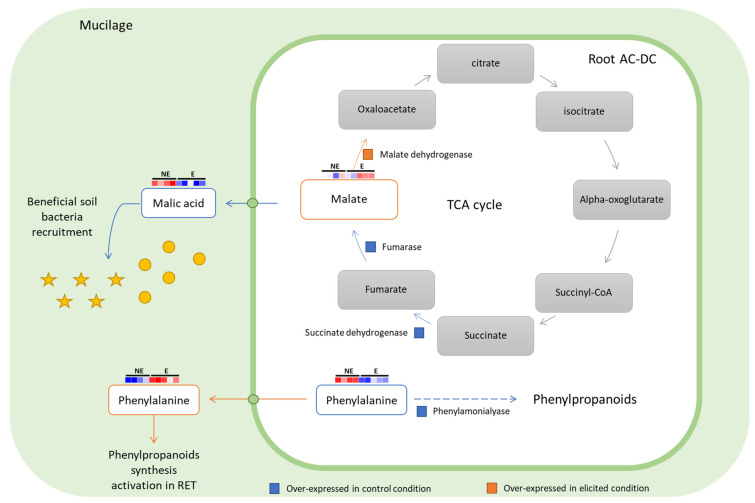
Malate and phenylalanine use in root AC-DC and mucilage is modified upon PEP-13 elicitation. The tricarboxylic acid (TCA) cycle in root AC-DC seems to be modified in control conditions (NE: non-elicited) in order to transfer malic acid in the mucilage. Upon elicitation with PEP-13 (E), a malate deshydrogenase is over-expressed, enabling the conversion of malate in oxaloacetate during the TCA cycle. In control conditions, phenylalanine is found in root AC-DC along with an over-expressed phenylamonialyase. These two actors might induce the synthesis of phenylpropanoids in root AC-DC. Upon elicitation, phenylalanine is found in mucilage and might be used as a defense signal for other cells and roots by activating their phenylpropanoid synthesis. Yellow circles and stars represent beneficial soil bacteria.

Among primary metabolites, malic acid seems to be secreted into mucilage in control conditions. Upon elicitation, malic acid found in mucilage decreases, and slightly increases in root AC-DC samples. In cells, malate is involved in the tricarboxylic acid cycle (TCA), which produces energy via oxidative processes [91]. Our transcriptomic data also show differentially expressed genes involved in the TCA cycle (Figure 6). A succinate dehydrogenase gene (GLYMA_06G305600) and a fumarase gene (GLYMA_02G015800) were found to be under-expressed upon elicitation, and a malate dehydrogenase gene (GLYMA_07G185400) was found to be over-expressed upon elicitation. Succinate dehydrogenase and fumarase enzymes are responsible for fumarate (malate precursor in TCA cycle) and malate synthesis. In control conditions, root AC-DC seems to produce high levels of malate, which is then delivered into the mucilage. Malate dehydrogenase enzyme is responsible for oxaloacetate synthesis from malate. As in elicited conditions this enzyme is over-expressed and malic acid quantity in mucilage decreases, it seems that upon elicitation, the secretion of malate is stopped, and it might be redirected to the TCA cycle for energy production. According to this hypothesis, the malic acid released into mucilage might play a role in the RET. Indeed, malic acid is able to recruit beneficial bacteria such as *Bacillus subtilis* FB17, as shown in the study by Rudrappa et al. in 2008 [92]. In that study, Arabidopsis roots were responsible for malic acid secretion upon the pathogen *Pseudomonas syringae* pv *tomato* infection. Here, this defense response was found to be constitutively activated in root AC-DC (Figure 6). 

The primary metabolite phenylalanine was detected among the VIP > 2 metabolites. In root AC-DC, phenylalanine was mainly found in non-elicited conditions and seems to decrease significantly in elicited conditions. In mucilage, an opposite phenomenon was observed: phenylalanine was found at high levels in elicited conditions. In non-treated conditions, the high amount of phenylalanine combined with over-expression of a phenylalanine ammonia-lyase (PAL) gene (GLYMA_03G181700) found in our transcriptomic results suggest that root AC-DC produce secondary metabolites. Indeed, deamination of L-phenylalanine by PAL leading to the formation of trans-cinnamate is the first step of the phenylpropanoid biosynthesis pathway [88,89]. This hypothesis is also supported by the high number of phenylpropanoids that are found to be over-represented in root AC-DC in control conditions (14 with VIP > 2 in Figure 5). The use of phenylalanine release in mucilage by root AC-DC upon elicitation was investigated. It has been shown that external application of phenylalanine on Arabidopsis, petunia and tomato plants [93] and even on mango and avocado fruits [94] induces resistance to fungal pathogens such as *Botrytis cinerea* Whetzel, 1945, *Colletotrichum gloeosporioides* (Penz.) Penz. & Sacc., 1884 and *Lasiodiplodia theobromae* (Pat.) Griffon & Maubl., 1909 via accumulation of phenylpropanoid compounds. Upon PEP-13 elicitation, root AC-DC might release phenylalanine in the mucilage in order to activate phenylpropanoid synthesis in the whole RET, leading to a more efficient defense response against fungi, and which might also be more efficient against other pathogens such as the oomycete *P. sojae*.

### 3.3. Deciphering the Basal and Induced Defense Responses in the RET

To provide an overview of the entire RET system upon basal and induced defense mechanisms to the scientific community, an original approach using multi-omics methods (transcriptomic, proteomic and metabolomic) was used on our control and PEP-13 elicited samples of roots, root AC-DC and mucilage from soybeans. Thus, this study provides new knowledge and a global vision of a still poorly understood compartment of plant roots: the RET and its response to PEP-13 elicitation. Our major finding is the transfer of metabolites from root AC-DC to mucilage upon PEP-13 elicitation.

**Figure 7:**
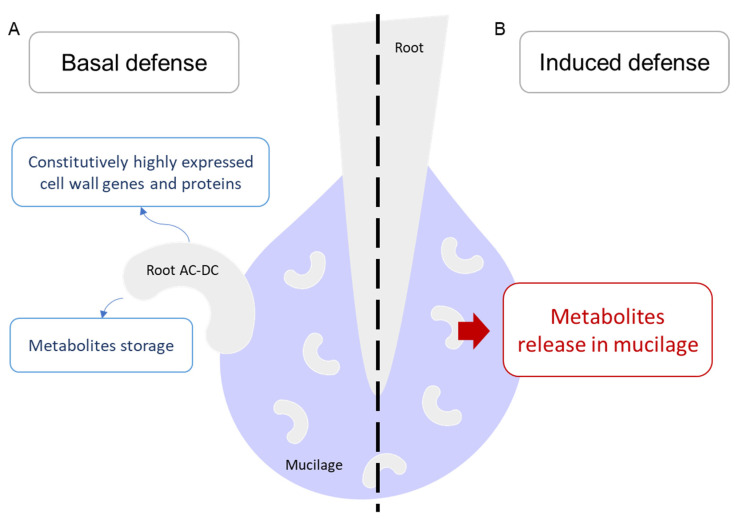
Overview of root AC-DC basal and induced defense responses. (**A**) In root AC-DC, constitutive differences with roots mainly consist of highly expressed cell wall genes and proteins and metabolite storage. (**B**): Upon PEP-13 elicitation, a transfer of metabolites was observed from root AC-DC to the mucilage.

Briefly, our analysis showed the presence of a basal defense in the RET (highly expressed cell wall genes and metabolite accumulation in root AC-DC) and a defense response induced by PEP-13 elicitation, mainly consisting of metabolite release in the mucilage (Figure 7). To our knowledge, our study is the first one to combine three high-throughputs methods (transcriptomic, proteomic and metabolomic) on the whole RET, progressing beyond the state-of-the-art. 

So far, the RET has been described as a structure that has two major types of action: (I) recruitment of beneficial organisms and (II) repulsion, attraction and immobilization of pathogens [5]. In order to achieve these functions, the literature has described the reorganization of root AC-DC cell wall components as extensines upon elicitation [28,29]. The mucilage composition: polysaccharides [39], specialized metabolites [40,95], proteins [96] and extracellular DNA [38,41] was investigated. The mucilage was also found to be organized as a network structure [63] and as a defensive structure with the constitutive presence of defensins [97], chitinases [96] and pisatin [37] in the RET of various plants. 

Our data also showed a constitutive defense response involving cell wall genes and proteins that were over-expressed in root AC-DC compared to root samples (AGPs, expansines, cellulose synthases and pectinesterases). We observed the constitutive occurrence of three proteins over-expressed in root AC-DC compared to root samples that were involved in response to biotic stimulus (Gene onthology 9607, Uniprot), including a chitinase (glyma19g43470.1) and two uncharacterized proteins corresponding to a defensin-like protein (Uniprot blast 90% identity: A0A1S3TEI1 locus LOC106754639, *Vigna radiata var. radiata*) and a PR-5 protein (Uniprot blast 90% identity: B6ZHC0 locus GLYMA_01G217700, *Glycine max*). 

Furthermore, genes and proteins over-expressed upon PEP-13 elicitation were involved in defense response, including PTI activation, cell wall and mucilage reinforcement and DAMP production were observed. However, the number of differentially expressed proteins was not high between elicited and constitutive conditions. In order to explain this observation, a time-related explanation was hypothesized. Indeed, our samples were collected with the same protocol for each experiment (transcriptomic, proteomic and metabolomic), so the exposure time to the PEP-13 elicitor was the same for gene, protein and metabolite experiments (5 days). This timing allowed us to microscopically observe a response of the RET—an accumulation of root AC-DC (data not shown)—as shown previously in the literature upon elicitation and infection of plants [37,39]. Nevertheless, it may be possible that a first response to PEP-13 has already occurred, and most RNA transcripts and proteins might already have been degraded. 

Another hypothesis concerning the low number of differentially expressed proteins might be the use of post-translational modifications (PTM) upon defense response [98], which could not be detected with our proteomic method. Indeed, although the Mascot database is able to recognize PTM as peptides with cysteine carbamidomethyl and methionine oxidation, our analysis was not designed to detect these modifications. Thus, undetected PTM in our data might be involved in PEP-13 elicitation response. In the literature, high dephosphorylation of some extracellular proteins was shown on elicited maize cell cultures [99], indicating that phosphorylation and dephosphorylation might be involved in the defense response. Indeed, our transcriptomic results showed 28 kinase genes (11 over-expressed upon PEP-13 elicitation) and 11 phosphatases genes (5 over-expressed upon PEP-13 elicitation), and our proteomic results showed 1 protein phosphatase under-expressed upon PEP-13 elicitation; thus, phosphorylation PTM might be an important regulatory process upon induced defense response. 

Our metabolomic analysis showed an interesting result: the compartment changes from root AC-DC to mucilage upon elicitation of a high number of secondary metabolites, mainly phenylpropanoids. Given these results, it can be deduced that root AC-DC store metabolites, including flavonoids, in control conditions; then, these metabolites are secreted upon elicitation. According to the literature, ABC and MATE transporters are involved in flavonoid secretion [100].

Differentially expressed transporters between root AC-DC and root samples were compared. In non-treated conditions, 19 ABC transporters are over-expressed in roots compared to root AC-DC, and only 6 are over-expressed in root AC-DC compared to roots. Upon elicitation of roots and root AC-DC, the number of ABC transporter genes differentially expressed increases, and 27 ABC transporters are over-expressed in roots compared to root AC-DC, and 13 are over-expressed in root AC-DC compared to roots. 

As flavonoid transport seems more important in roots than in root AC-DC, flavonoids might be produced by roots cells and transported to peripheral cells before their detachment, then stored in root AC-DC until their release upon induced defense response.

Our analysis also showed an interesting result on primary metabolism of root AC-DC that is still poorly described in the literature. Since a 1986 study showing that root AC-DC might be isolated then cultivated [101], these cells have mainly been studied for their secretions and defense responses [28,34,35,36,37,40,102,103].

Thanks to our metabolomic data, we observed that root AC-DC might deviate malate from the TCA cycle in control conditions to secrete it in mucilage in order to use malic acid to recruit beneficial organisms. Upon elicitation, evidence showed that malate seemed to be reoriented to the TCA cycle to produce energy. This hypothesis was strengthened by transcriptomic results showing the over-expression of genes leading to malate synthesis in control conditions, and the over-expression of a gene involved in the conversion of malate into oxaloacetate upon elicitation. Such mechanisms have not yet described in the literature.

## 4. Conclusions

The major finding of this study is the change of compartment of specialized metabolites from root AC-DC to the mucilage upon elicitation with PEP-13, a defense response that is different from roots. This study also provides high-throughput data generated on root AC-DC and roots at different scales of analysis (transcriptomic, proteomic and metabolomic). This new knowledge on the RET defense response might help the scientific community to develop new methods of biotic and abiotic stress management, new methods of mineral nutrition optimization and new knowledge of rhizosphere function; all of these will develop more-sustainable agriculture.

## Data Availability

Omics data created for this study are available in the ENA database under accession PRJEB52621.

## Supplementary Materials

The following supporting information can be downloaded at: https://www.mdpi.com/article/10.3390/cells11162605/s1, Figure S1: Supervised multivariate classification OPLS-DA of AC-DC, mucilage and roots with and without PEP-13 elicitation, related to metabolomic analysis. Table S1: Cell wall over-expressed genes and proteins in AC-DC compared to roots in control condition, related to Figure 2. Table S2: Differentially expressed genes and proteins upon PEP13 elicitation in roots, related to Figure 3. Table S3: Differentially expressed genes and proteins upon PEP13 elicitation in AC-DC, related to Figure 3.
