# Supplementary material for: Elicitation of Roots and AC-DC with PEP-13 Peptide Shows Differential Defense Responses in Multi-Omics
Source: Cells. 2022 Aug 21;11(16):2605. doi: 10.3390/cells11162605 (PMC9406913; doi:10.3390/cells11162605)
Supplement: Supplementary file 1 [file cells-11-02605-s001.zip › 220720 Data supp.pdf]

Table S1: Cell wall over-expressed genes and proteins in AC-DC compared to roots in control condition, related to figure 2.
See excel file, sheet "Table S1". The column "Bin name" corresponds to gene ontologies obtained with Mapman. ID = identifier. Bold identifiers correspond to proteins. (fold change ≥ 1,5; p-value ≤ 0,05)

Table S2: Differentially expressed genes and proteins upon PEP13 elicitation in roots, related to figure 3.
See excel file, sheet "Roots E vs NE". (fold change ≥ 1,5; p-value ≤ 0,05)

Table S3: Differentially expressed genes and proteins upon PEP13 elicitation in AC-DC, related to figure 3.
See excel file, sheet "AC-DC E vs NE". (fold change ≥ 1,5; p-value ≤ 0,05)

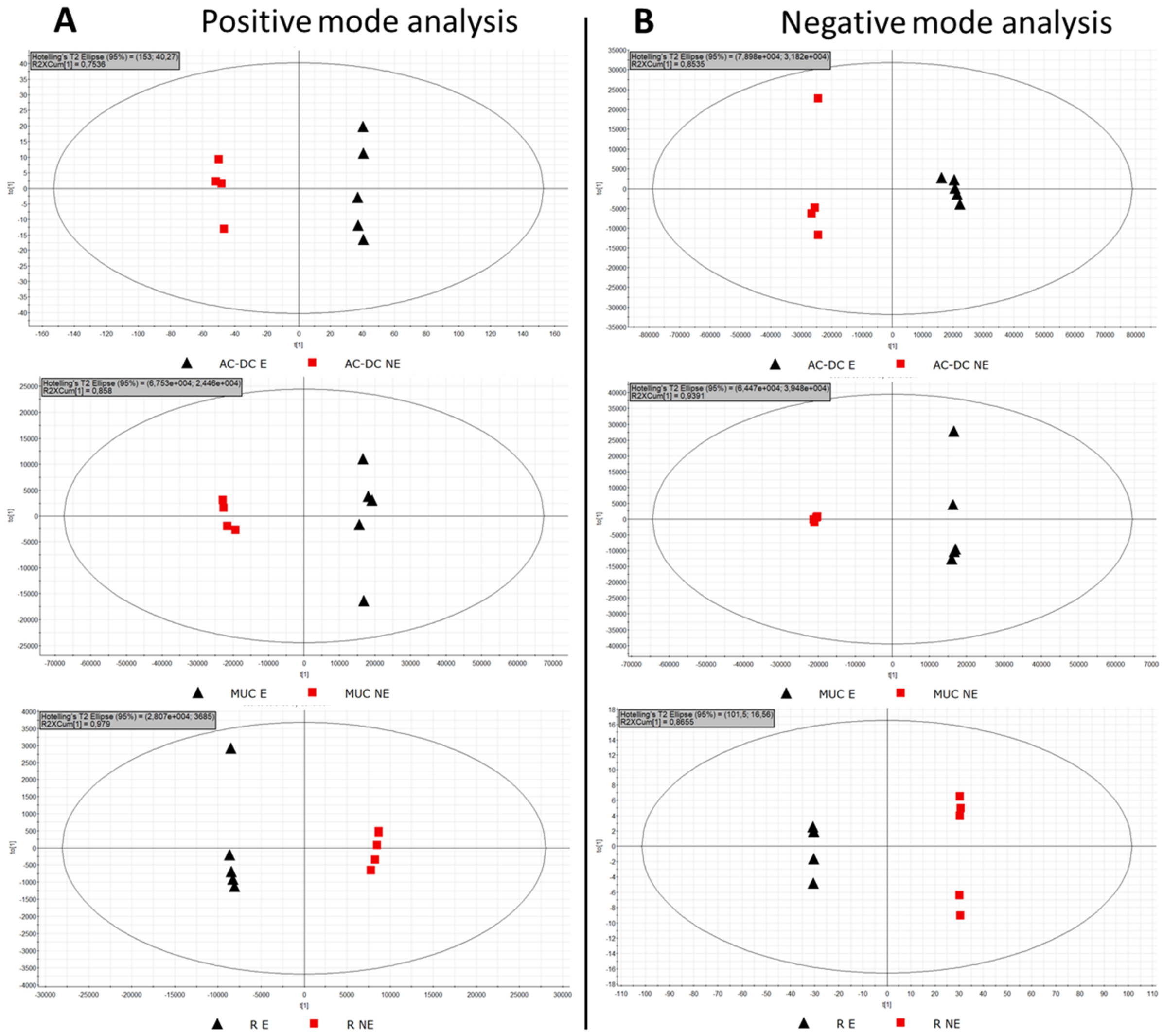

Figure S1: Supervised multivariate classification OPLS-DA of AC-DC, mucilage and roots with and without PEP-13 elicitation, related to metabolomic analysis.
R =roots; MUC = mucilage; AC-DC = root Associated Cap-Derived Cells.
